# Monocyte Chemoattractant Protein-1 promotes cancer cell migration via c-Raf/MAPK/AP-1 pathway and MMP-9 production in osteosarcoma

**DOI:** 10.1186/s13046-020-01756-y

**Published:** 2020-11-23

**Authors:** Ju-Fang Liu, Po-Chun Chen, Tsung-Ming Chang, Chun-Han Hou

**Affiliations:** 1 School of Oral Hygiene, College of Oral Medicine, Taipei Medical University, Taipei, 110 Taiwan; 2 Department of Medical Research, China Medical University Hospital, China Medical University, Taichung, 40447 Taiwan; 3 Translational medicine center, Shin-Kong Wu Ho-Su Memorial Hospital, Taipei City, 11101 Taiwan; 4 Department of Biotechnology, College of Medical and Health Science, Asia University, Taichung, 41354 Taiwan; 5 School of Medicine, Institute of Physiology, National Yang-Ming University, Taipei City, 11221 Taiwan; 6 Department of Orthopedic Surgery, National Taiwan University Hospital, 100, NO. 1, Jen-Ai Road, Taipei City, 11102 Taiwan, ROC

**Keywords:** Osteosarcoma, MCP-1, Migration, MMP-9

## Abstract

**Background:**

Osteosarcoma is generally reported among younger individuals and has a very poor prognosis, particularly for the development of metastasis. However, more effective metastatic biomarkers and therapeutic methods are absent. Monocyte chemoattractant protein-1 (MCP-1) is involved in cancer progression and inflammatory recruitment. Although previous studies have reported higher serum MCP-1 levels in patients with osteosarcoma, the role of MCP-1 in osteosarcoma progression remains to be addressed.

**Methods:**

The osteosarcoma cell migratory ability was assessed by transwell migration assay. The MCP-1 and MMP-9 expression levels were analyzed by Western blot and qPCR. The signal activation was conducted by Western blot. The in vivo mouse experiment and tumor tissue array were performed to confirm our findings in vitro.

**Results:**

The present study demonstrates that MCP-1 regulates cell mobility through matrix metalloproteinase (MMP)-9 expression in osteosarcoma cells. Moreover, MCP-1 promotes MMP-9 expression, cell migration, and cell invasion by mediating CCR2, c-Raf, MAPK, and AP-1 signal transduction. Using MCP-1 knockdown stable cell lines, we found that MCP-1 knockdown reduces MMP-9 expression and cell mobility. Finally, we found high MCP-1 expression levels in osteosarcoma specimens.

**Conclusions:**

Our results provide prognostic value of MCP-1 in osteosarcoma by promoting MMP-9 expression.

**Supplementary Information:**

The online version contains supplementary material available at 10.1186/s13046-020-01756-y.

## Background

Osteosarcoma is the most common primary bone malignancy, and it accounts for 30 to 80% of primary skeletal sarcomas [1]. Osteosarcoma diagnoses are classified into four grades according to the histological degree of diffusion and differentiation; higher grades indicate more aggressive malignant neoplasms [2]. High-grade osteosarcoma represents the development of metastasis, mainly in the lungs [3]. Osteosarcoma metastasis also occurs in organs such as the bones and lymph nodes [4]. Pulmonary metastasis can be observed in approximately 15–20% of the patients at initial diagnosis and in 40% of the patients at a later follow-up [5]. Metastasis is one of the leading causes of poor prognosis in patients with osteosarcoma; only 20% of the patients survive for more than 5 years [6]. An increasing number of promising cytokines and biomarkers have been identified for preventing osteosarcoma metastasis or improving therapeutic outcomes [7]. However, the detailed pathological mechanisms and the ideal treatment method for osteosarcoma are not fully understood.

Metastasis involves cancer cell detachment, invasion, intravasation, and extravasation [8]. Matrix metalloproteinases (MMPs), a family of zinc- and calcium-dependent proteolytic enzymes, can facilitate the extracellular matrix to dissolve and regulate the expression of other cytokines [9]. MMPs are involved in biological processes such as inflammation, cell differentiation, proliferation, angiogenesis, apoptosis, and migration [10]; they also participate in tumorigenesis and metastasis in numerous types of cancer cells [11, 12]. Recently, MMP-1, MMP-2, MMP-7, MMP-12, MMP-13, and MMP-26 have been reported to be associated with lung cancer progression, invasion and metastasis [13–16]. Additionally, MMP-2, MMP-9, and MMP-13 overexpression has been revealed to be a promising indicator for osteosarcoma prognosis and pulmonary metastasis [17–19]. However, MMPs are complicated, and their roles in osteosarcoma development are mostly unknown.

Chemokines are categorized into four categories, namely CXC, CC, CX3C, and C, on the basis of their cysteine residues at the N-terminus [20]. Monocyte chemoattractant protein-1 (MCP-1/CCL2), a key member of the CC chemokine family, is associated with inflammatory diseases and cancers, including ovarian [21], colon [22], and prostate cancers [23] etc. MCP-1 is produced by endothelial cells, smooth muscle cells, fibroblasts, and monocytes [24, 25] constitutively or through stimulations. In the tumor microenvironment, MCP-1 is overexpressed by both tumor and nontumor cells including stromal cells [26]. MCP-1 regulates the receptor of intracellular adhesion molecule-1 (ICAM-1) to facilitate monocyte adhesion [27]. In addition, MCP-1 affects macrophage infiltration and migration and angiogenesis in gastric cancer [28]. The monocytes induced by MCP-1 can promote lung metastasis in breast cancer [29]. Moreover, tumor cell growth, motility, invasion, and metastasis are associated with MCP-1 in different types of cancer including hepatocellular carcinoma [30]. A previous study demonstrated that MCP-1 promotes osteosarcoma migration and proliferation through the Akt pathway [31]. However, the mechanism through which MCP-1 stimulates metastasis remains unclear.

In the present study, we found that MCP-1 exhibit metastasis-promoting roles by increasing MMP-9 expression in osteosarcoma. MCP-1 expression level was tightly associated with migratory potential in osteosarcoma cells. Using molecular and pharmacological strategies, we found that MPC-1 enhanced MMP-9 expression and cell migration through CCR2, c-Raf, MAPK signal pathway and AP-1 transcriptional activation. Our finding here provides a novel insight into the molecular mechanisms of metastasis in osteosarcoma which could be a novel therapeutic target in the future.

## Methods

### Materials

All shRNA plasmids with specific targets, and control shRNA plasmid were purchased from National RNAi Core Facility (Academia Sinica, Taipei, Taiwan). Protein G beads; all secondary antibodies against rabbit or mouse (IgG-conjugated horseradish peroxidase); rabbit polyclonal antibodies against β-actin, MMP-2, MMP-3, MMP-9, MMP-12, MMP-13, c-Raf, p-MEK, MEK, p-ERK, ERK, p-JNK, JNK, p-p38, p38, p-c-Jun, c-Jun were obtained from Santa Cruz Biotechnology (Santa Cruz, CA, USA). All siRNAs which target specific genes (si-c-Jun: sc-29,223; si-MMP9: sc-29,400; si-CCR2: sc-270,220; si-control: sc-44,231) were purchased from Santa Cruz Biotechnology. p-c-Raf (Ser338) was purchased from Cell Signaling (Danvers, MA, USA). U0126 and PD98059 were purchased from Calbiochem (San Diego, CA, USA).

The dominant-negative mutants specific for MEK1, ERK2, JNK and p38 were kindly gifted from Dr. W. M. Fu (National Taiwan University, Taipei, Taiwan), Dr. M. Cobb, (University of Texas Southwestern Medical Center, Dallas, TX, USA), Dr. M Karin (University of California, San Diego, CA, USA), and Dr. J. Han (University of Texas Southwestern Medical Center, Dallas, TX, USA), respectively. Recombinant human MCP-1/CCL2 was purchased from PeproTech (Rocky Hill, NJ, USA). AP-1 luciferase plasmid was purchased from Stratagene (La Jolla, CA, USA). The remaining chemicals used were purchased from Sigma–Aldrich (St. Louis, MO, USA).

### Cell culture

All osteosarcoma cell lines (U2OS, HOS, and MG-63) and normal osteoblast cell line (hFOB 1.19) used in the current study were obtained from American Type Cell Culture Collection (Manassas, VA, USA). U2OS cells were maintained in McCoy’s 5A medium. MG63, HOS, and hFOB 1.19 cells were maintained in DMEM medium. To make a complete medium, the other components were added according to ATCC’s description. Furthermore, the cells were cultured in the presence of antibiotics (100 U/mL of penicillin and 100 μg/mL of streptomycin) at 37 °C and 5% CO_2_.

### Migration assay

Transwell migration assay was conducted with Transwell plate (Costar, NY; pore size, 8 μm). Briefly, 300 μL of the serum-free medium was added in the lower chamber with different concentrations of MCP-1. Meanwhile, 100 μL of serum-free medium contained 1 × 10^4^ cells were added in the upper chamber. The cells were incubated at 37 °C in 5% CO_2_ for 24 h. After 24 h later, the transwell inserts were fixed in 3.7% formaldehyde for 15 min. Then, 0.05% crystal violet dissolved in PBS was added to stain the cells for 15 min. The Transwell inserts were washed with PBS, and the cells in the upper chamber were removed using cotton swabs. The cells which migrated to the lower side of the Transwell inserts were further observed and counted using a microscope. For each experimental condition, a minimum of three experiments were conducted in triplicate.

### Establishment of migration-prone subclones from osteosarcoma cell line

The MG63 (M10, M20 and M30) migration-prone subclones were established by using Transwell inserts (6 wells plate with 8 μm pore size). MG63 osteosarcoma cells (1 × 10^4^) suspended in 100 μL of serum-free medium were seeded in the upper chamber, while 300 μL growth medium contained 10% FBS was loaded into the lower compartment. After 24 h later, the cell which migrated across the Transwell insert to the bottom of plate were detached by trypsin and cultured as MG63 (M1). The cells were cultured for 2 days for a second round of selection. The MG63 migration-prone subclone was continued migration seletion for 10, 20, 30 rounds to generate MG63 (M10), MG63 (M20) and MG63 (M30), respectively.

### Wound healing migration assay

Each of the 12 wells was filled with 1 × 10^5^ cells, and the plate was incubated for 24 h. The confluent monolayer of cultured cells was scratched using a fine pipette tip. The rate of wound closure was recorded and calculated through microscopic observation. For each experimental condition, a minimum of three experiments were conducted in triplicate.

### Western immunoblot analysis

Protein expression was examined using a Thermo Scientific Pierce BCA Protein Assay Kit (Thermo Fisher Scientific Inc., Waltham, USA). Sodium dodecyl sulfate (SDS)-polyacrylamide gel electrophoresis was conducted to resolve proteins in cell lysate, followed by transferred to Immobilon polyvinylidene difluoride (PVDF) membranes. Next, to block the blots, PVDF membranes were incubated in 4% BSA at room temperature for 1 h. The PVDF membranes were further incubated with primary antibodies for 1 h at room temperature, then washed three times with TBST. The membranes were later incubated with HRP-conjugated secondary antibodies for another 1 h at room temperature follow by washed with TBST for three times. The membranes were then detected by chemiluminescent substrate (Amersham™ ECL™ Western Blotting Detection Reagents; GE Healthcare Life Sciences, Marlborough, MA, USA) and monitored by using a charge-coupled device camera-based detection system (UVP Inc., Upland, CA, USA). The data were quantitatized by using ImageJ software (National Institute of Health, USA).

### Quantitative real-time PCR

The total RNA was extracted by using the TRIzol kit (MDBio Inc., Taipei, Taiwan) according to the manufacture’s protocol. Next, for reverse transcription into cDNA, 2 μg of total RNA was reacted with an oligo(dT) primer. TaqMan® one-step PCR Master Mix (Applied Biosystems, Foster City, CA, USA) was used for analysis by using quantitative real-time polymerase chain reaction (qPCR). After reverse transcription, the cDNA (100 ng/25 μL per-reaction) was further added with primers and TaqMan® probes for detecting specific sequences, as well as TaqMan Universal PCR Master Mix according to the manufacturer’s instructions. The polymerase activation cycle was 10 min at 95 °C, 15 s at 95 °C for 40 cycles, and finally 60 s at 60 °C. For each experimental condition, a minimum of three experiments were conducted in triplicate with a StepOnePlus sequence detection system.

### Transfection and reporter gene assay

The cells with 80 % confluency were co-transfected with AP-1-luciferase vector (0.8 μg) and a β-galactosidase expression vector (0.4 μg) for 24 h by using Lipofectamine 2000 (LF2000; Invitrogen, Carlsbad, CA, USA). The plasmids and LF2000 were mixed and incubated for 20 min and subsequently added to the cells for 24 h, followed by incubated with the indicated agents for another 24 h. Before the cells were washed with cold PBS, the media were first removed. To lyse the cells, 100 μL of reporter lysis buffer (Promega, Madison, WI, USA) was added, and the supernatant was collected. In addition, luciferase substrate was added to 20 μL of lysates with 20–30 μg protein. Luminescence was recorded using a microplate luminometer. The luciferase activity was evaluated after normalizing the cells with the cotransfected β-galactosidase expression vector. For each experimental condition, a minimum of three experiments were conducted in triplicate.

### Nuclear and cytoplasmic fractionation assay

The cells grown in 10-cm dishes were treated with MCP-1 for the indicated conditions and the cells were washed two times with ice-cold PBS, followed by scraped in PBS and collected cells by centrifuged for 15 mins. Subsequently, ice-cold buffer 1 was added (10 mM HEPES, pH 7.9; 10 mM KCl; 0.1 mM EDTA, pH 8; 0.1 mM EGTA, pH 8; 10 mM PMSF, 10 mM DTT, 10 mM NaF, 10 mM Na3VO4). The cells were sheared mechanically with a syringe and needle, then samples were centrifuged and the supernatant, i.e., the cytoplasmic fraction, was collected. The remaining pellet was washed 3 times with buffer 1, and then re-suspended in buffer 2 (20 mM HEPES, pH 7.9; 400 mM NaCl; 1 mM EDTA, pH 8; 1 mM EGTA, pH 8; 10 mM PMSF, 10 mM DTT, 10 mM NaF, 10 mM Na3VO4) and centrifuged; the supernatant was collected as the nuclear protein fraction.

### Chromatin immunoprecipitation assay

Chromatin immunoprecipitation analysis was conducted as previously described [32]. DNA was immunoprecipitated using anti-c-Jun mAb, and it was further purified. The DNA was extracted by adding phenol–chloroform. The purified DNA pellet was used for PCR. After the PCR reaction, products were resolved using 1.5% agarose gel electrophoresis. Next, UV light was used for visualization. The primers 5′- ATCCTGCTTCAAAGAGCCTG-3′ and 5′-GTCTGAAGGCCCTGAGTGGT-3′ were used for amplification across the human MMP-9 promoter region (− 547 to − 327).

### Establishment of MCP-1 knockdown stable cell lines

The MCP-1 and control shRNA lentiviral constructs (pLKO.1) were obtained from the National RNAi Core Facility (Academia Sinica, Taipei, Taiwan). The control shRNA (sh-control: ASN0000000004, target sequence: CCTAAGGTTAAGTCGCCCTCG) and the shRNAs that targeted MCP-1 (sh-MCP-1-A: TRCN0000006279, target sequence: GATGTAAACATTATGCCTTA; sh-MCP-1-B: TRCN0000006283, target sequence: CCCAGTCACCTGCTGTTATAA; and sh-MCP-1-C: TRCN0000381382, target sequence: TCATAGCAGCCACCTTCATTC) were purchased from the National RNAi Core Facility (Taipei, Taiwan).

The HEK293T cells were used to prepare lentivirus. Briefly, the shRNA plasmid mixed with packaging vectors pCMV and pMDG were co-transfected into HEK293T cells. After 24 h and 48 h post-transfection, the cell culture supernatants were collected and stored at -80 °C. To establish knockdown stable clones, the MG63 cells were transduced with cell culture supernatants describe above in the presence of 8 μg/ml of polybrene (Sigma–Aldrich). After 48 h post-transduction, the cells which expressed shRNA vectors were selected by the culture medium contained puromycin (10 μg/ml). Finally, the shRNA expression stable clones were generated after 2 weeks of selection with puromycin. In addition, all experiments used at least 2 distinct shRNA.

### In vivo metastasis model

All in vivo experiments were conducted according to Guidelines for Animal Care of the Institutional Animal Care and Use Committee of Shin-Kong Wu Ho-Su Memorial Hospital (Taipei, Taiwan) (Ethical approval No: Most1040002). The mice were purchased from the Lasco (Taipei, Taiwan). The 5-weeks male CB17-SCID mice were intravenous tail injected with osteosarcoma cells (2 × 10^6^ / 100 μL). Six weeks after tumor implantation, the mice were sacrificed and lungs were collected to analyze metastatic nodules. The lung tissues were fixed by 10% formalin and then embedded in paraffin and subsquently performed with hematoxylin and eosin (HE) staining.

### Immunohistochemistry

Human osteosarcoma tissue microarrays (BO244, T261, T262, T262A, T263, and OS804b) were obtained from Biomax (Rockville, MD, USA). All tissue microarray contained normal bone tissue (11 cases), stage I osteosarcoma (7 cases), stage II osteosarcoma (49 cases), and stage III osteosarcoma (7 cases). The paraffin-embedded tissues (5-μm thick) were rehydrated and incubated in 3% hydrogen peroxide to suppress endogenous peroxidase activity. Next, 3% bovine serum albumin (BSA) was prepared and used to block the samples, and it was subsequently replaced with phosphate-buffered saline (PBS) for incubation. The samples were further incubated at 4 °C with a primary mouse polyclonal antihuman antibody. After overnight incubation, the samples were washed with PBS. After three washes, the samples were incubated with a secondary antibody labeled with biotin. An ABC Kit purchased from Vector Laboratories (Burlingame, CA, USA) was used to detect the bound antibodies. Next, the samples were stained with chromogen diaminobenzidine. After another wash, the samples were stained with Delafield’s hematoxylin. Finally, the samples were dehydrated, mounted, and observed at five different degrees (0 (negative), 1 (very weak), 2 (weak), 3 (moderate), 4 (strong), and 5 (very strong)) of independent and blinded observations. The total intensity score was obtained from five immunohistochemistry (IHC) scores.

### Statistics

The values are represented as means ± the standard deviation (SD). Significant differences between the experimental groups and controls were assessed using the Student’s *t* test. Overall survival analysis was performed through the Fisher LSD post hoc tests. The differences in overall survival of the two groups were compared using the log-rank test; *p* < 0.05 was considered statistically significant.

## Results

### MCP-1-induced cell migration in osteosarcoma cell line can be further enhanced by MCP-1 supplementation

**Fig. 1:**
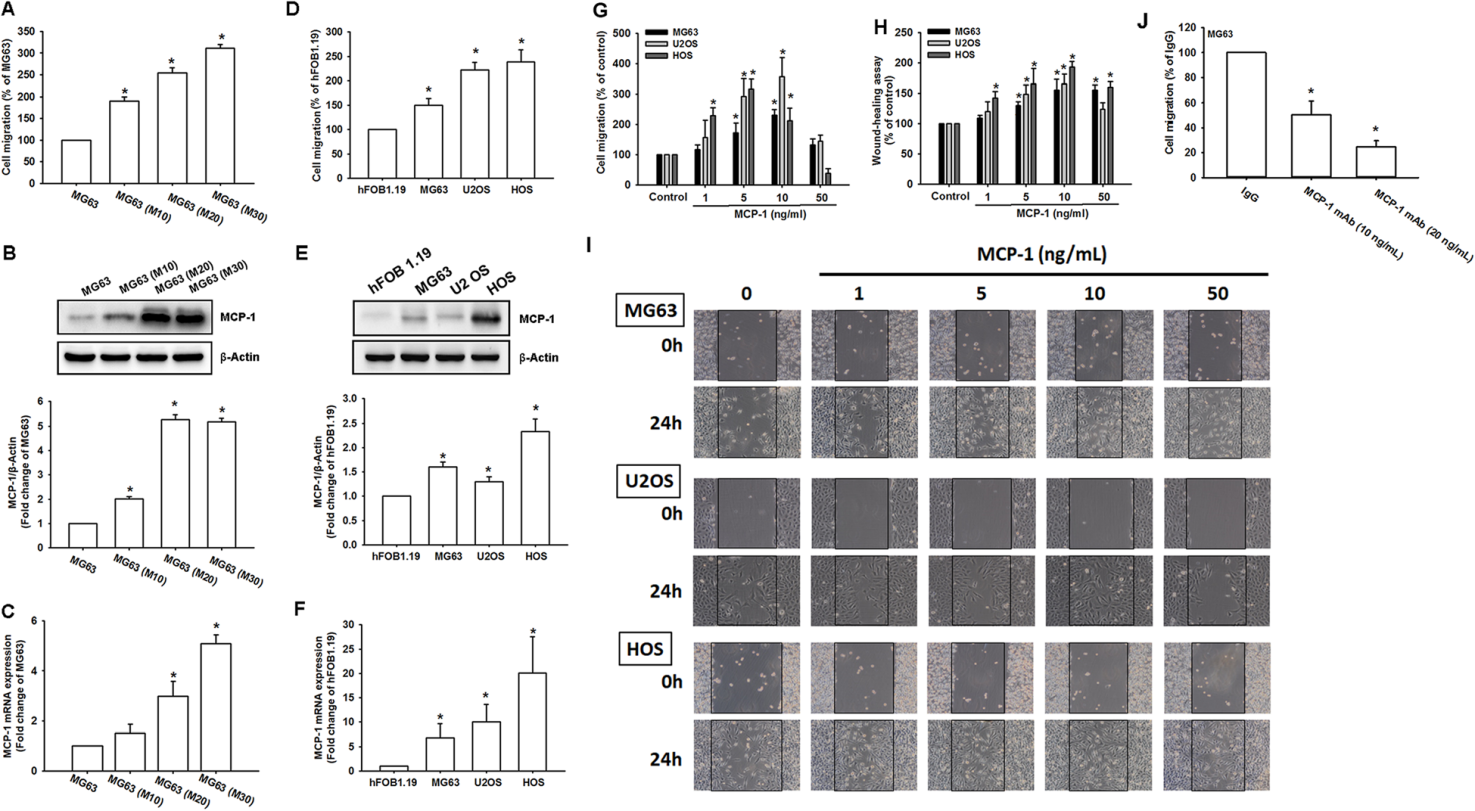
MCP-1 was involved in and promoted osteosarcoma migration. **a** A migration assay was performed in the MG63 cells with different migratory abilities (M10, M20, and M30). **b** MCP-1 protein production was detected in the MG63 cells with different migratory abilities (M10, M20, and M30) through Western blotting. **c** MCP-1 mRNA expression was compared between the MG63 cells with different migratory abilities (M10, M20, and M30) through a qPCR assay. **d** The cell migration ability of the osteoblast cell line hFOB 1.19 and the osteosarcoma cell lines MG63, U2OS and HOS was assessed using the Transwell assay. **e-f** Total mRNA and protein were collected from the indicated cell lines, and MCP-1 expression was detected using Western blotting and qPCR assay. **g-h** A migration assay and wound-scratching assay were performed, respectively, in the MG63, U-2OS, and HOS cells after stimulation with different concentrations of MCP-1 (1, 5, 10, and 50 ng/mL). **i** Representive image of wound-scratching assay in Fig. 1h. **j** A migration assay was performed in the MG63 cells in response to different concentrations of MCP-1 mAb (10 and 20 ng/mL). Results are expressed as mean ± SEM, *n* = 4. *p < 0.05 compared with MG63 (Fig. 1a-c), hFOB1.19 (Fig. 1d-f), control (Fig. 1g-h) and IgG (Fig. 1j), respectively

MCP-1 has been shown to increase cell migration and metastasis in various human cancer cells. To understand the effect of MCP-1 on osteosarcoma cells, we first selected and cultured an osteosarcoma cell line, MG63, with different degrees of migratory ability including 10, 20, and 30 generations and compared their migratory efficiency (Fig. 1a). The higher the generation was, the more the cells could migrate. Consequently, we detected the MCP-1 protein (Fig. 1b) and mRNA (Fig. 1c) expression among different selected cells. MCP-1 protein and mRNA production both increased the most during the 30 generation MG63 cells. Meanwhile, the association between MCP-1 and osteosarcoma cell migration potential was confirmed in osteosarcoma cell lines including MG-63, U2OS, HOS as well as normal osteoblast cell line hFOB 1.19 (Fig. 1d-f), which was in agreement with our findings in migration-prone cells above. Of the different concentrations of MCP-1, the MG63, U2OS and HOS cells stimulated with 10 ng/mL of MCP-1 exhibited the highest migratory degrees (Fig. 1g). In the HOS cells, the highest migratory ability was observed for stimulation with less than 5 ng/mL of MCP-1. In the wound healing ability test, 10 ng/mL of MCP-1 triggered the highest degrees of migration in the three osteosarcoma cell lines (Fig. 1h and i). When two different concentrations of MCP-1 antibody were used in the MG63 cells, the original migratory effect could be significantly reduced (*p* < 0.05) (Fig. 1j). Therefore, MCP-1 production was highly correlated with osteosarcoma cell migration in vitro.

### MMP-9 was involved in MCP-1-mediated osteosarcoma cell migration

**Fig. 2:**
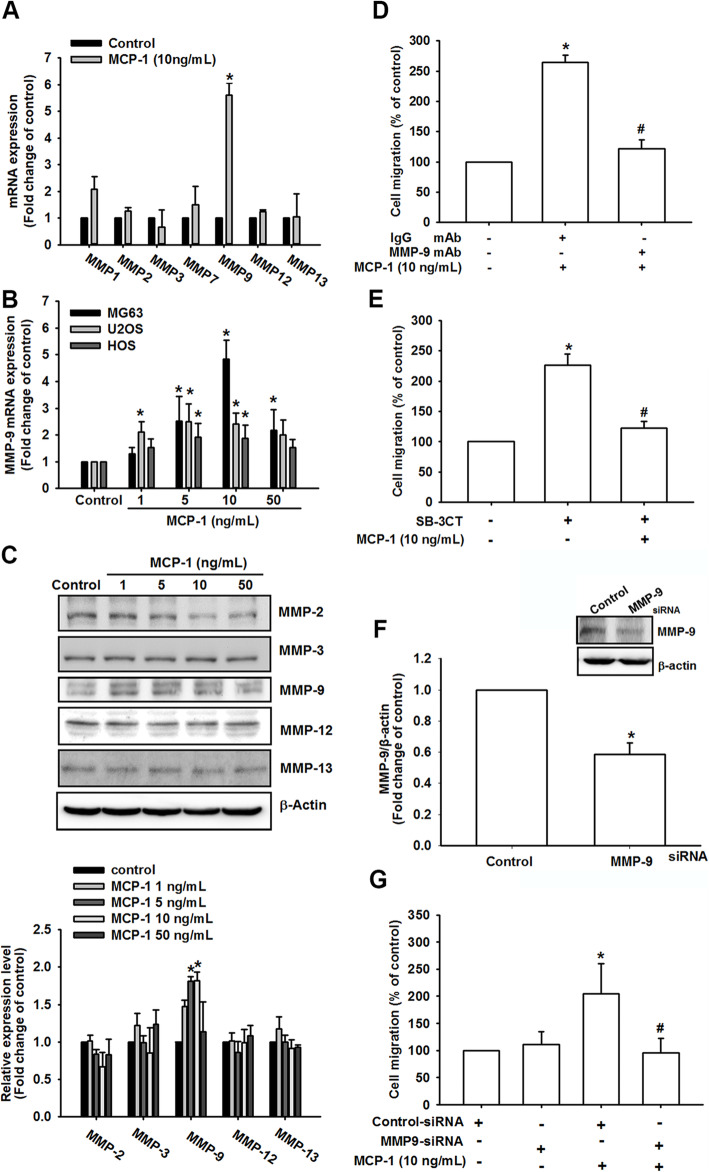
MMP-9 was involved in MCP-1-mediated osteosarcoma migration. **a** MMP-1, MMP-2, MMP-3, MMP-7, MMP-9, MMP-12, and MMP-13 mRNA expression were measured after 10 ng/mL MCP-1 for 24 h stimulation through a qPCR assay. **b** MMP-9 mRNA expression was measured in the MG63, U-2OS, and HOS cells after stimulation with different concentrations of MCP-1 (1, 5, 10, and 50 ng/mL) for 24 h. **c** MMP-2, MMP-3, MMP-9, MMP-12, and MMP-13 expression were observed with stimulation at different concentrations of MCP-1 (1, 5, 10, and 50 ng/mL) for 24 h through a Western blotting assay. **d** MG-63 cells were treated with control and MMP-9 mAb for 30 min, and cell migration ability was measured with MCP-1 (10 ng/mL) for 24 h stimulation. **e** MMP-9 inhibitor (SB-3TC; 10 μM) was added to the MG63 cells, and cell migration ability was measured with MCP-1 (10 ng/mL) for 24 h stimulation. **f**-**g** Control and MMP-9 siRNA were first transfected into the MG63 cells and incubated for 24 h, MMP-9 protein expression and cell migration ability were measured with or without MCP-1 stimulation. Results are expressed as mean ± SEM, n = 4. *p < 0.05 compared with control, IgG or control siRNA groups; #*p* < 0.05 compared with the MCP-1-treated group

Studies have revealed that MMPs including MMP-1, MMP-2, MMP-3, MMP-7, MMP-9, MMP-12, and MMP-13 are significantly related to osteosarcoma metastasis and poor prognosis [19, 33–38]. To identify the mediator of MCP-1-promoted osteosarcoma migration, we further examined the expression of MMP-1, MMP-2, MMP-3, MMP-7, MMP-9, MMP-12, and MMP-13 mRNA under MCP-1 stimulation (Fig. 2a). The results revealed that substantial amounts of only MMP-9 mRNA were produced after MCP-1 treatment. In addition, MMP-9 mRNA was upregulated in a dose-independent manner (Fig. 2b). Western blotting demonstrated that among the expression levels of MMP-2, MMP-3, MMP-9, MMP-12, and MMP-13 only that of MMP-9 increased in a dose-dependent manner (Fig. 2c and Fig. S2). The expression levels of MMP-9 among migration-prone subclone, osteosarcoma cell lines and normal osteoblasts were also evaluated (Fig. S1, upper panel), and our results indicated that MCP-1 expression was positively associated with MMP-9 expression. When osteosarcoma cells were treated with MMP-9 monoclonal antibody (mAb), the degree of cell migration significantly decreased (Fig. 2d). Moreover, with MMP-9 inhibitor (SB-3CT), less osteosarcoma cell migration was demonstrated (Fig. 2e). In addition to pharmacological inhibition, we also used MMP-9 small interfering RNA (siRNA) to confirm the migratory effect (Fig. 2f-g). MCP-1-promoted osteosarcoma cell migratory ability remarkably decreased after MMP-9 siRNA pretreatment. Therefore, MCP-1 triggered osteosarcoma metastasis through the regulation of MMP-9 expression.

### MCP-1 promoted osteosarcoma migration through the CCR2 receptor

**Fig. 3:**
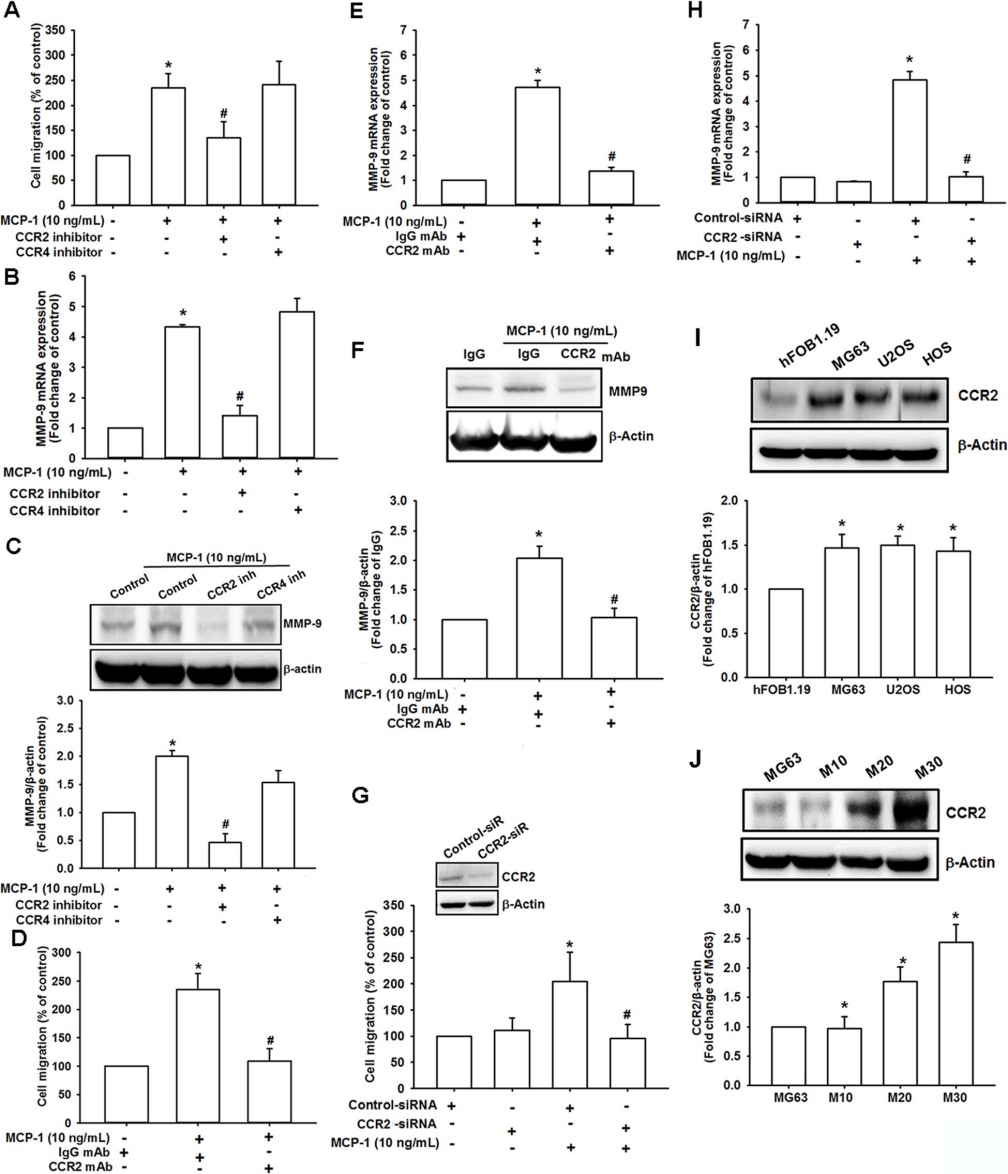
MCP-1-induced osteosarcoma migration required the CCR2 receptor. **a** CCR2 and CCR4 inhibitors (400 nM) were added to the cells. After MCP-1 stimulation, MG63 migration was measured. **b** and **c** MMP-9 mRNA and protein expression were detected, respectively, with CCR2 and CCR4 inhibitor treatment and MCP-1 stimulation. (D and E) After the MG63 cells were treated with CCR2 mAb for 30 min, cell migration and MMP-9 mRNA expression were measured, respectively. **f**-**h** The MG63 cells were transfected with CCR2 siRNA for 24 h, and the CCR2 protein expression, cell migration and MMP-9 mRNA expression were measured with or without MCP-1 stimulation. **i** CCR2 protein production was detected in the osteoblast cell line hFOB 1.19 and the osteosarcoma cell lines MG63, U2OS and HOS were assessed using Western blotting. **j** CCR2 protein production was detected in the MG63 cells with different migratory abilities (M10, M20, and M30) through Western blotting. Results are expressed as mean ± SEM, *n* = 4. *p < 0.05 compared with control, IgG, control siRNA, hFOB1.19 or MG63 groups, respectively; #*p* < 0.05 compared with the MCP-1-treated group

CCR2, one of the most important MCP-1 receptors, regulates MCP-1 effects more strictly than MCP-1 itself [39]. A study of 27 patient samples with osteosarcoma revealed CCR2 expression in every sample [40]. However, in another study, CCR4 was found in 21 out of 22 osteosarcoma samples [40]. Consequently, we examined whether MCP-1 induced MG63 osteosarcoma cell migration through the CCR2 or CCR4 receptor by using pharmacological inhibitors. In the migration assay, osteosarcoma cell migration was remarkably reduced by the CCR2 inhibitor but not by the CCR4 inhibitor (Fig. 3a). MMP-9 mRNA expression was also substantially inhibited by the CCR2 inhibitor but not by the CCR4 inhibitor after MCP-1 stimulation (Fig. 3b). In addition, MMP-9 protein production was reduced with CCR2 inhibitor treatment (Fig. 3c and Fig. S3). We further used CCR2 mAb to examine the MCP-1-mediated MG63 cell migratory effect (Fig. 3d). With CCR2 mAb, MCP-1-induced migration was drastically reversed. Moreover, pretreatment with CCR2 mAb demonstrated a similar effect as the CCR2 inhibitor on MMP-9 expression level in response to MCP-1 stimulation (Fig. 3e-f). In addition to the CCR2 inhibitor and mAb, we used CCR2 siRNA to confirm the involvement of CCR2 in MCP-1-promoted osteosarcoma cell migration. CCR2 siRNA could reverse both the cell migration degree (Fig. 3g) and the production of MCP-1-mediated MMP-9 mRNA (Fig. 3h). Finally, we defined the expression level of CCR2 among osteosarcoma cell lines and normal osteoblast cell line, which may be associated with migration ability in these cell lines (Fig. 3i). As expected, the positive correlation was found between CCR2 expression level and migration ability among these cell lines (Fig. 3j), suggesting that CCR2 was closely related to MCP-1-induced osteosarcoma cell metastasis as a key receptor.

### C-Raf signaling participated in MCP-1-mediated osteosarcoma migration

**Fig. 4:**
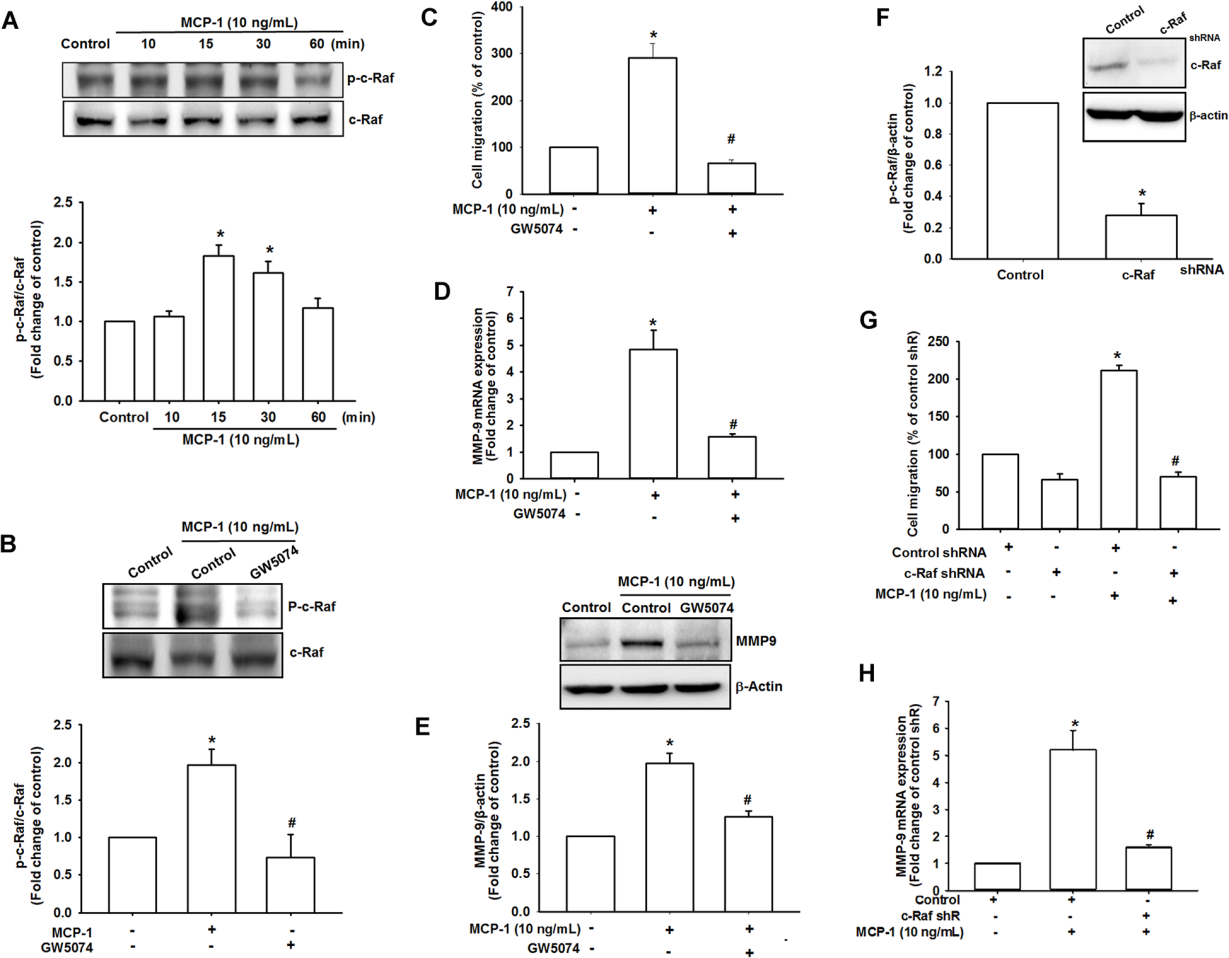
c-Raf was involved in MCP-1-mediated osteosarcoma migration. **a** At different MCP-1 stimulation durations (0, 10, 15, 30, and 60 min), c-Raf phosphorylated protein and c-Raf total protein were measured. **b** MG63 cells were pretreated with 0.1% DMSO as a control, GW5074 for 30 min and then incubated with MCP-1 for 30 min. c-Raf phosphorylation was examined using Western blotting. **c** The MG63 cells were treated with GW5074 (5 μM), and the degree of cell migration was measured with MCP-1 stimulation. **d** and **e** MMP-9 mRNA and protein expression were detected after the MG63 cells were treated with GW5074 and MCP-1 stimulation. **f**-**g** After the control shRNA and c-Raf shRNA were transfected in the MG3 cells and incubated for 24 h, a cell migration assay was performed with or without MCP-1 stimulation. **h** The MG63 cells were transfected with c-Raf shRNA and incubated for 24 h, and MMP-9 mRNA expression was measured using the qPCR assay. Results are expressed as mean ± SEM, *n* = 4. *p < 0.05 compared with control or control shRNA groups; #*p* < 0.05 compared with the MCP-1-treated group

Previous studies have demonstrated that c-Raf is important in the regulation of epithelial to mesenchymal transition, which is an essential process of tumor invasion and metastasis in numerous types of cancer, including osteosarcoma and chondrosarcoma [32, 41–43]. Under MCP-1 stimulation, western blotting demonstrated that phosphate c-Raf was activated in a time-dependent manner (Fig. 4a). Pretreatment with c-Raf inhibitor (GW5074) for 30 min markedly inhibited MCP-1-induced phosphorylation of c-Raf (Fig. 4b). Pharmacologically, the MG63 cells treated with GW5074 demonstrated a drastically decreased migratory ability (Fig. 4c and Fig. S3) as well as MMP-9 expression (Fig. 4d and e). Finally, to further examine whether c-Raf was involved in MCP-1-mediated osteosarcoma migration, we used the c-Raf short hairpin RNA (shRNA) (Fig. 4f). When the MG63 cells were transfected with c-Raf shRNA, both cell migration (Fig. 4g) and MMP-9 mRNA expression (Fig. 4h) were remarkably reversed. According to these results, MCP-1 induced MMP-9 expression and cell migration through the c-Raf signaling pathway in osteosarcoma.

### MCP-1-induced migration involved the MAPK signaling pathway

**Fig. 5:**
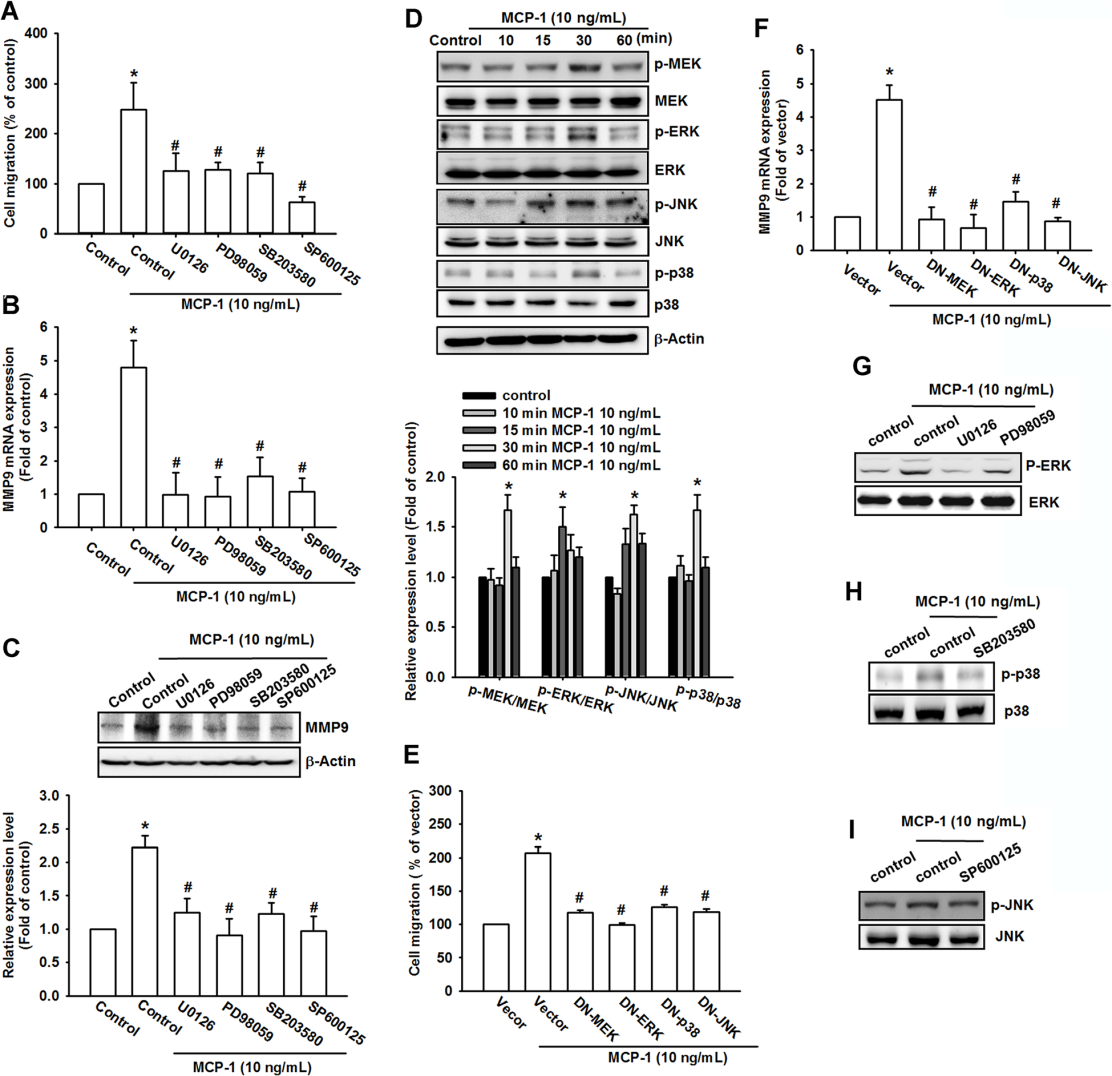
MCP-1-promoted osteosarcoma migration involved the MAPK signaling pathway. **a** The MG63 cells were first treated with U0126 (10 μM), PD98059 (10 μM), SB203580 (10 μM), and SP600125 (10 μM), and a cell migration assay was performed with MCP-1 stimulation. **b** and **c** After pretreatment with different inhibitors (U0126, PD98059, SB203580 and SP600125) and MCP-1 stimulation, MMP-9 mRNA and protein production were measured, respectively. **d** At different MCP-1 stimulation durations (0, 10, 15, 30, and 60 min), MEK, ERK, p38, and JNK phosphorylated and total proteins were measured using Western blotting. **e** and **f** The MG63 cells were transfected with MEK, ERK, p38, and JNK dominant negative (DN) mutants for 24 h, and the degree of cell migration and MMP-9 mRNA were measured using qPCR. **g**-**i** MG63 cells were pretreated with 0.1% DMSO as a control, ERK inhibitor (U0126), MEK inhibitor (PD98059)¸ JNK inhibitor (SP600125) or p38 inhibitor (SB203580) for 30 min and then incubated with MCP-1 for 30 min. p38, JNK and ERK phosphorylation were examined using Western blotting. Results are expressed as mean ± SEM, n = 4. *p < 0.05 compared with control or vector groups; #*p* < 0.05 compared with the MCP-1-treated group

The c-Raf/MEK/ERK signaling cascade has been reported to be related to tumorigenesis and metastasis [32, 43, 44]. Studies have revealed that MEK, ERK, JNK, and p38 MAPK play crucial roles in MMP-9 regulation and osteosarcoma metastasis [45]. Therefore, we examined whether MEK, ERK, JNK, and p38 MAPK were also important mediators in MCP-1-mediated MG63 cell migration. We used different pharmacological antagonists, including MEK inhibitors (U0126 and PD98059), p38 MAPK inhibitor (SB203580), and JNK inhibitor (SP600125) to demonstrate the influences on MCP-1-promoted MG63 cell migration (Fig. 5a). MMP-9 production and MCP-1-mediated MG63 cell migratory ability were drastically reduced by the antagonists (Fig. 5b and Fig. S3). Western blotting revealed that MMP-9 mRNA expression was decreased, and MMP-9 protein production was inhibited (Fig. 5c). To determine whether MEK, ERK, JNK, and p38 MAPK were activated during MCP-1-triggered cell migration, each protein phosphorylation was measured using western blotting (Fig. 5d). In addition, we used MEK, ERK, JNK, and p38 MAPK mutants to examine the cell migratory effect (Fig. 5e) and MMP-9 mRNA regulation (Fig. 5f). With different methods of antagonists, MEK, ERK, JNK, and p38 MAPK mutants also remarkably reversed the effects of MCP-1 upregulation. Pretreatment with MEK inhibitors (U0126 and PD98059), p38 MAPK inhibitor (SB203580), and JNK inhibitor (SP600125) for 30 min markedly inhibited MCP-1-induced phosphorylation of ERK, JNK, and p38 MAPK (Fig. 5g-i). Therefore, MAPK is crucial in osteosarcoma metastasis promoted by MCP-1 upregulation.

### MCP-1 regulated AP-1-activation MMP-9 overexpression through CCR2/c-Raf/MAPK signaling pathway

**Fig. 6:**
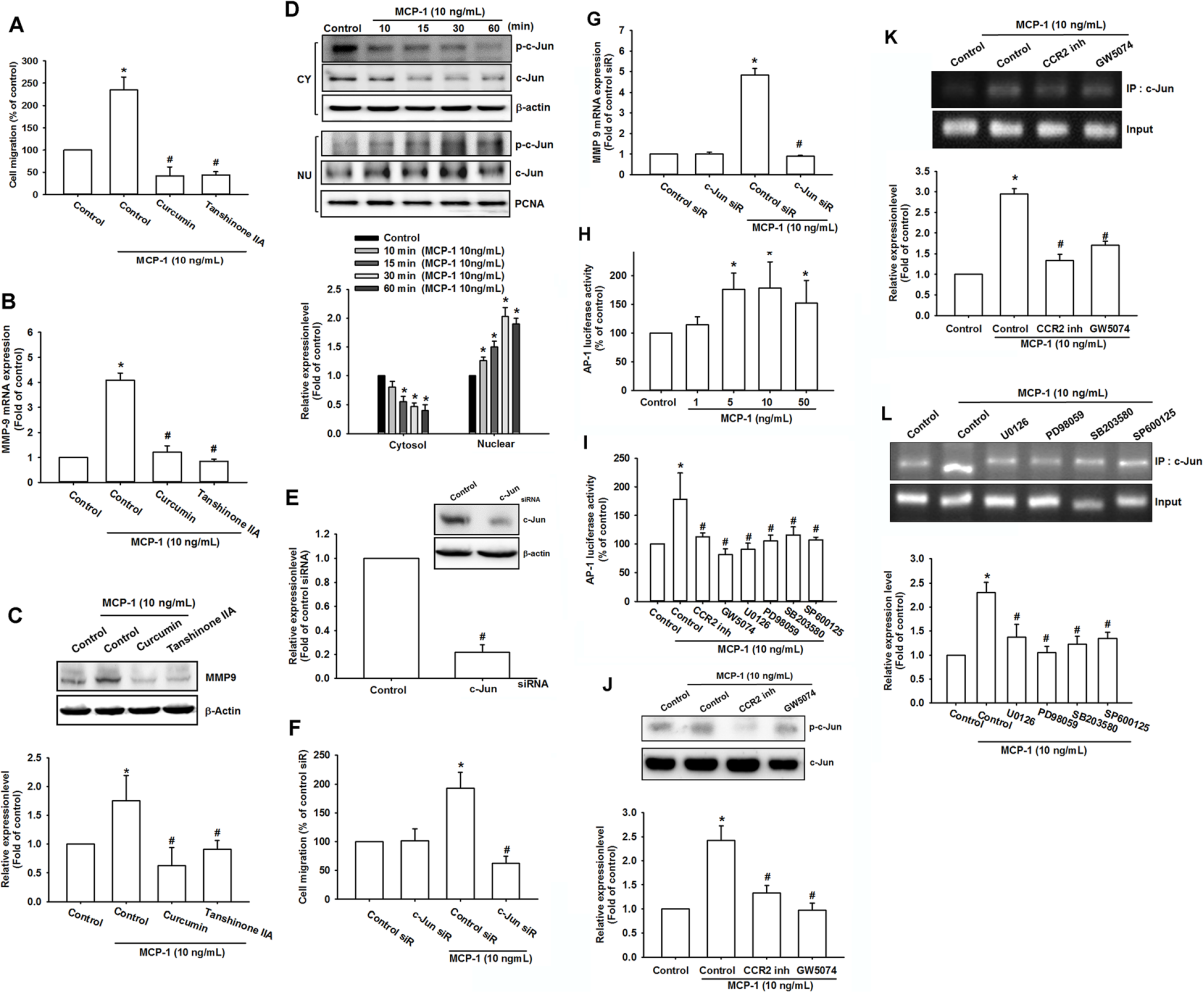
MCP-1-promoted osteosarcoma migration and MMP-9 upregulation required c-Jun/AP-1 involvement. **a** A migration assay was performed in the MG63 cells with curcumin (1 μM) and tanshinone IIA (5 μM) pretreated for 30 min with MCP-1 stimulation. **b** and **c** The MG63 cells were treated with curcumin and tanshinone IIA for 30 min, and MMP-9 mRNA and protein expression were measured. **d** At different MCP-1 stimulation durations (0, 10, 15, 30, and 60 min), c-Jun phosphorylated and total proteins in both the cytosol and nucleus were measured using Western blotting. **e** The MG63 cells were transfected with control and c-Jun siRNA and then incubated for 24 h. A migration assay was performed to measure osteosarcoma migratory ability. **f**-**g** The MG63 cells were transfected with control and c-Jun siRNA and incubated for 24 h. Cell migration and MMP-9 mRNA expression was measured with MCP-1 stimulation. **h** AP-1 luciferase activity was measured in the MG63 cells at various concentrations of MCP-1 stimulation. The results were normalized according to the β-galactosidase activity. **i** The MG63 cells were treated with MCP-1 stimulation and different inhibitors (CCR2 inhibitor, GW5074, U0126, PD98059, SB203580, and SP600125), and AP-1 luciferase activity was measured and normalized according to the β-galactosidase activity. **j** The MG63 cells were treated with the CCR2 inhibitor and GW5074 for 30 min, respectively, and c-Jun phosphorylated and total protein production were measured using Western blotting. **k** and **l** After the MG63 cells were pretreated with the CCR2 inhibitor, GW5074, U0126, PD98059, SB203580, and SP600125 for 30 min, MCP-1 was added to stimulate the cells for 120 min. The chromatin immunoprecipitation assay was then performed with anti-c-Jun immunoprecipitation. To verify equal loading amount (input), 1% of the precipitated chromatin was applied. Results are expressed as mean ± SEM, n = 4. *p < 0.05 compared with control or control siR groups; #*p* < 0.05 compared with the MCP-1-treated group

AP-1, an important binding site of c-Jun and c-Fos, has been reported to regulate numerous types of gene transcription, such as MMPs [46]. Studies have revealed the critical role of c-Jun in osteosarcoma progression and metastasis [47, 48]. Therefore, we hypothesized that AP-1 was also involved in MCP-1-mediated migration. Through the pharmacological blocking of the c-Jun function (curcumin and tanshinone IIA), substantially fewer MG63 cells migrated than through MCP-1 stimulation only (Fig. 6a and Fig. S3). Additionally, both curcumin and tanshinone IIA remarkably reduced MMP-9 expression in qPCR (Fig. 6b) and the Western blotting assay (Fig. 6c). Next, we examined c-Jun activation and transportation during the MCP-1-induced migration in osteosarcoma cells. Western blotting demonstrated that c-Jun was phosphorylated in the cytosol only, and the total expression of c-Jun was detected in the nucleus (Fig. 6d). In addition to pharmacological antagonists, c-Jun functional siRNA was used. c-Jun siRNA did not exert a noticeable effect on MG63 cell migration; however, it could drastically reverse the effects of MCP-1 on MG63 cell (Fig. 6e-f). As presented in Fig. 6g, MMP-9 mRNA expression was also remarkably reduced even after MCP-1 stimulation. Therefore, AP-1 was an essential mediator in MCP-1-promoted MMP-9 upregulation and osteosarcoma migration.

AP-1 activation has been reported to regulate MMP-9 transcription and cancer invasion [46, 49]. Figure 6g and h present the AP-1 luciferase activity. MCP-1 activated AP-1 in a dose-dependent manner (Fig. 6h). The CCR2 inhibitor, GW5074, U0126, PD938059, SB203580, and vSP600125 drastically reduced the MCP-1-mediated migration in the MG63 cells (Fig. 6i). In addition, western blotting demonstrated that the CCR2 and c-Raf antagonists reduced c-Jun phosphorylation (Fig. 6j). The chromatin immunoprecipitation assay revealed that the binding of c-Jun to the AP-1 segment regulating the MMP-9 expression was increased after treatment with MCP-1 (Fig. 6k). By contrast, pretreatment with the CCR2 inhibitor and c-Raf inhibitor resulted in less AP-1 binding and MMP-9 transcription (Fig. 6l). U0126, PD98059, SB203580, and SP600125 also attenuated MCP-1-promoted AP-1 binding effects (Fig. 6l). Taken together, MCP-1-promoted AP-1 activation and MMP-9 expression through the CCR2, c-Raf, MAPK, and c-Jun pathways.

### MCP-1 expression is associated with metastasis in an animal model in osteosarcoma

**Fig. 7:**
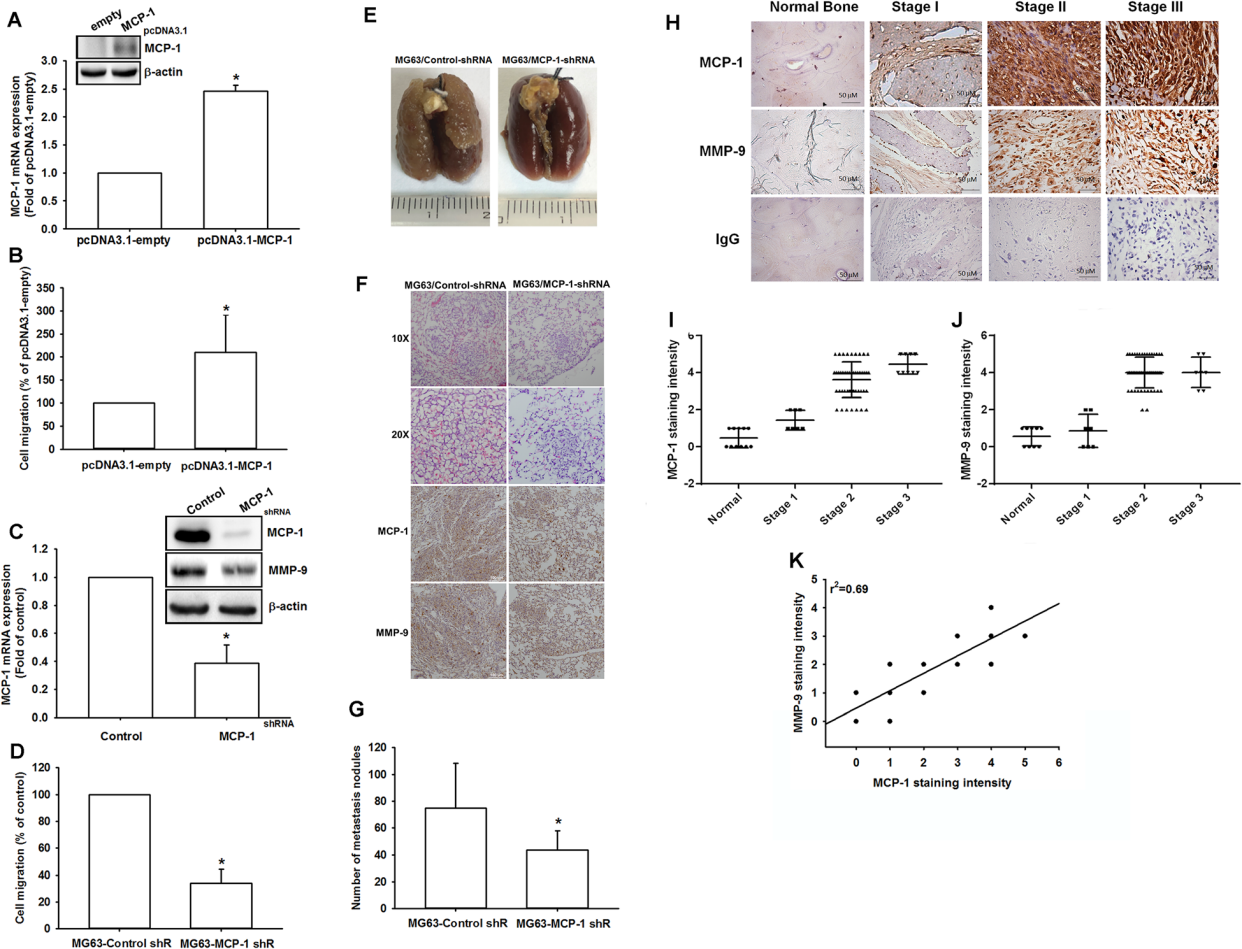
Knockdown of MCP-1 inhibited the migratory ability of osteosarcoma cells. **a**-**b** MCP-1-overexpressing MG-63 cells were established, using pCDNA3.1 vector. Protein and mRNA expression of MCP-1 and migratory activity were examined by western blot, q-PCR, and Transwell assay. **c** Treating MG63 cells with shRNA against MCP-1 decreased the protein level of MCP-1 as well as that of MMP-9. **d** shRNA knockdown of MCP-1 decreased the in vitro migration activity of osteosarcoma cells. **e** To induce pulmonary metastases, MG63 cells were injected into the mouse tail vein and those mice were sacrificed after 28 days later with developed lung metastatic nodules. Compared to the control mice, there are fewer and smaller tumors which were seen on the lungs of mice injected with osteosarcoma cells transfected with shRNA against MCP-1. **f** H&E staining and IHC of lung metastatic nodules of MG63 injected mice. **g** The lungs of MG63 injected mice were removed and inflated with 10% paraformaldehyde fixative. The number of lung metastatic nodules was counted under a dissecting microscope. **h** MCP-1 and MMP-9 expression levels in specimens were determined in a tumor tissue microarray by IHC staining. The stained specimens were photographed using an optical microscope. **i** and **j** IHC stain intensities were scored from 1 to 5 to quantify MCP-1 and MMP-9 expression levels in different stages. **k** Correlation between MCP-1 and MMP-9 expression in human osteosarcoma specimens. Results are shown as mean ± SEM. n = 4. *p < 0.05 compared with pcDNA3.1-empty, control or MG63-control shR groups

The in vitro results in the current study showed that MCP-1 contributes to cancer cell migration by inducing MMP-9 expression in osteosarcoma cells. Therefore, we conducted the osteosarcoma cells (MG63) which stably expressed MCP-1 or MCP-1 shRNA, which were further subjected to confirm MCP-1 expression levels and migration potential (Fig. 7a-d). Also, the in vivo animal study was performed with MG63 cells stably expressed MCP-1 shRNA or control vector by intravenous injection into the tail vein. Twenty eight days after implantation with cancer cells, the mice were sacrificed and lung nodules were monitored to evaluate metastatic potential of stable clones. The data indicated that lung nodules were dramatically abolished in MG-63/MCP-1-shRNA stable clone (Fig. 7e and g). Meanwhile, in hematoxylin and eosin (H&E) staining and IHC results, the lung morphology in mice implanted with MG-63/MCP-1-shRNA stable clone exhibited decreased degrees of lung metastatic nodules, while control group mice were infiltrated with metastases (Fig. 7f). These evidence indicated that MCP-1 promotes metastasis of osteosarcoma in vivo.

### MCP-1 is highly expressed and correlated with prognosis in osteosarcoma patient specimens

Our findings indicate that MCP-1 induces MMP-9 expression in osteosarcoma cells. To confirm these findings, we examined the expressions of MCP-1 and MMP-9 in different-stage osteosarcoma cells as well as in normal human bone tissue. The tissue array results revealed that higher concentrations of MCP-1 and MMP-9 were produced as the high grade of osteosarcoma increased (Fig. 7h). Compared with the normal bone tissue, stage II osteosarcoma cells exhibited 2–3 times higher MCP-1 expression, and stage III osteosarcoma cells exhibited 3–4 times higher MCP-1 production (Fig. 7i). The MMP-9 expression in stage II and stage III osteosarcoma cells was 2–3 and 3–4 times, respectively, higher than that in normal tissue (Fig. 7j). Furthermore, we investigated the correlation between MCP-1 and MMP-9 upregulation. The correlation coefficient of MCP-1 and MMP-9 expression was 0.69 (Fig. 7k). These results indicate that MCP-1 expression is correlated with MMP-9 expression, tumor progression, and prognosis in patients with osteosarcoma.

## Discussion

Unlike other bone malignancies, osteosarcoma is difficult to diagnose early and effectively cure [6]. The long-term survival rate for osteosarcoma is very low, particularly for metastasis cases [6]. The study findings suggest that the pulmonary metastasis of osteosarcoma is highly related to MCP-1 upregulation. Here, we examined the efficacy of MCP-1 knockdown on osteosarcoma metastasis, which showed inhibition of pulmonary metastasis. This evidence provide opportunity to implicate MCP-1 as a new potential therapeutic direction in human clinical studies of osteosarcoma. In regard to biological nature of MCP-1, the major chemoattractant of macrophage, its roles in tumor microenvironment of osteosarcoma by affecting macrophages have been summarized in previous review [50]. The bone-specific macrophages (osteoclasts) have been implicated in osteosarcoma metastasis, which is achieved by blocking the vicious cycle between osteosarcoma cells and osteoclasts in tumor microenvironemnt [51]. Furthermore, bisphosphonates show promosing anti-tumoral effect by supressing osteosarcoma-mediated osteolysis, which is mainly caused by decreased secretion of MCP-1 in osteosarcoma cells [52]. These evidence propose that MCP-1 secreted by osteosarcoma cells could promote metastasis by modulating macrophages function in tumor microenvironment. However, the detail mechanism should be investigated in the future.

This study revealed the downstream activator of MCP-1-regulated osteosarcoma migration. MMPs have been widely proven to be essential in the process of cancer growth, invasion, angiogenesis, and metastasis [11]. Studies have revealed that MMPs such as MMP-2, MMP-3, and MMP-9 are involved in osteosarcoma progression and metastasis [19, 53, 54]. Interestingly, high MMP-9 expression is associated with poor prognosis in patients with osteosarcoma in several meta-analysis reports [55–57]. This evidence is in accordance with our finding in clinical specimens in osteosarcoma. Furthermore, MCP-1 only mediated MMP-9 production rather than other MMPs. We also found positive correlation between MCP-1 and MMP-9 in migration-prone subclones (Fig. S1A) of osteosarcoma as well as in tumor tissue array of osteosarcoma patients. These evidence suggest tight correlation between MCP-1 and MMP-9. One study found that MCP-1 can induce cancer cell migration through the upregulation of MMP-9 in chondrosarcoma [43]. Based on previous studies, MMP-9 might be an essential factor for MCP-1-induced metastasis in bone malignancies. Therefore, we hypothesized that MMP-9 are key mediators in MCP-1-induced osteosarcoma migration. Our findings implied that MMP-9 attenuation might be a new therapeutic target for osteosarcoma metastasis.

G protein-coupled receptors of MCP-1, including CCR2 and CCR4, mediate various biological functions [58, 59]. The functions of CCR2 differ according to different cell surfaces [39]. On antigen-presenting cells and T cells, CCR2 stimulates inflammatory effects; on T regulatory cells, CCR2 inhibits inflammation [39]. A recent study revealed that both CCR2 and CCR4 are highly expressed and involved in patients with osteosarcoma [40]. In this study, CCR2 instead of CCR4 was involved in osteosarcoma migration in vitro. Through the different methods of CCR2 antagonists, MCP-1-regulated MMP-9 production and metastasis were effectively reduced. This finding is consistent with that of a previous study, which demonstrated MCP-1/CCR2 axis regulation in chondrosarcoma migration [43]. Therefore, these findings are suggestive of a crucial role of CCR2 in MCP-1-mediated migration.

Ras, MEK, and MAPK signal transduction is commonly involved in cancer progression, including angiogenesis [60]. In addition, ERK, p38, and JNK are widely reported in osteosarcoma progression [61]. The result found the sustained JNK activation in response to MCP-1 incubation during 15–60 min. The MAPK signal proteins are activated in parallel, however, there is a crosstalk between them and modulates their activities [62]. Previous study has proved that JNK can switch from a transient to sustained activation state, which was regulated by ERK, p38, and AKT pathways. The switch of JNK activation state is associated with cell fates such as proliferation and apoptosis [63]. In osteosarcoma, the JNK signaling pathway is a critical component during metastasis process and has potential to develop as therapeutic target [64]. Our results reveal that JNK might be a key regulator in osteosarcoma metastasis in response to MCP-1 incubation.

In this study, we investigated the mechanism of MCP-1-regulated osteosarcoma migration, which remains unknown according to our review of the relevant literature. Our findings suggested that c-Raf, MAPK, and c-Jun were sequentially involved in osteosarcoma migration. This signal pathway regulated AP-1 activation and MMP-9 transcription, which further upregulated osteosarcoma migration. A previous study revealed that in chondrosarcoma, the MCP-1/CCR2 axis requires c-Raf, MEK, ERK signal pathways for MMP-9 overexpression [43]. However, the present study suggested that AP-1 played a crucial role in MCP-1/CCR2-directed metastasis in osteosarcoma.

## Conclusions

Among bone cancers with very poor prognoses, osteosarcoma is the most common. Therefore, effective diagnostic and therapeutic methods are urgently required. This study suggested that MCP-1 promoted osteosarcoma migration through the CCR2 receptor and the upregulation of MMP-9 expression through c-Raf, MAPK, c-Jun, and AP-1 activation. This signal pathway may help in elucidating the mechanism of osteosarcoma metastasis, which may provide a new direction toward developing more effective therapies in the future.

## Supplementary Information

**Additional file 1: Figure S1.** Expression levels of MMP9 and CCR4 in migration-prone subclones of MG63 osteosarcoma cells, osteosarcoma cell lines and normal osteoblasts. **Figure S2.** MPC-1 contributes to MMP-9 expression in osteosarcoma cell lines. **Figure S3.** MCP-1 promotes osteosarcoma cells migration through CCR2, c-Raf, MAPK and AP-1 signal pathways

## Data Availability

The data sets used and analyzed during the current study are available from the corresponding author on reasonable request.

## Abbreviations

MCP-1: Monocyte chemoattractant protein-1
MMPs: Matrix metalloproteinases
ICAM-1: Intracellular adhesion molecule-1
SDS: Sodium dodecyl sulfate
PVDF: Polyvinylidene difluoride
qPCR: Quantitative real-time polymerase chain reaction
BSA: Bovine serum albumin
PBS: Phosphate-buffered saline
SD: Standard deviation
mAb: Monoclonal antibody
siRNA: Small interfering RNA
shRNA: Short hairpin RNA
