# Supplementary material for: Monocyte Chemoattractant Protein-1 promotes cancer cell migration via c-Raf/MAPK/AP-1 pathway and MMP-9 production in osteosarcoma
Source: J Exp Clin Cancer Res. 2020 Nov 23;39:254. doi: 10.1186/s13046-020-01756-y (PMC7684958; doi:10.1186/s13046-020-01756-y)
Supplement: Supplementary file 1 — Additional file 1: Figure S1. Expression levels of MMP9 and CCR4 in migration-prone subclones of MG63 osteosarcoma cells, osteosarcoma cell lines and normal osteoblasts. Figure S2. MPC-1 contributes to MMP-9 expression in osteosarcoma cell lines. Figure S3. MCP-1 promotes osteosarcoma cells migration through CCR2, c-Raf, MAPK and AP-1 signal pathways [file 13046_2020_1756_MOESM1_ESM.docx]

**Supplementary information**

Title: Monocyte Chemoattractant Protein-1 promotes cancer cell migration via c-Raf/MAPK/AP-1 pathway and MMP-9 production in osteosarcoma

Authors: Ju-Fang Liu, Po-Chun Chen, Tsung-Ming Chang, and Chun-Han Hou* Hsun-Hsien Chang, Gon-Ann Lee and Chi-Feng Hung.
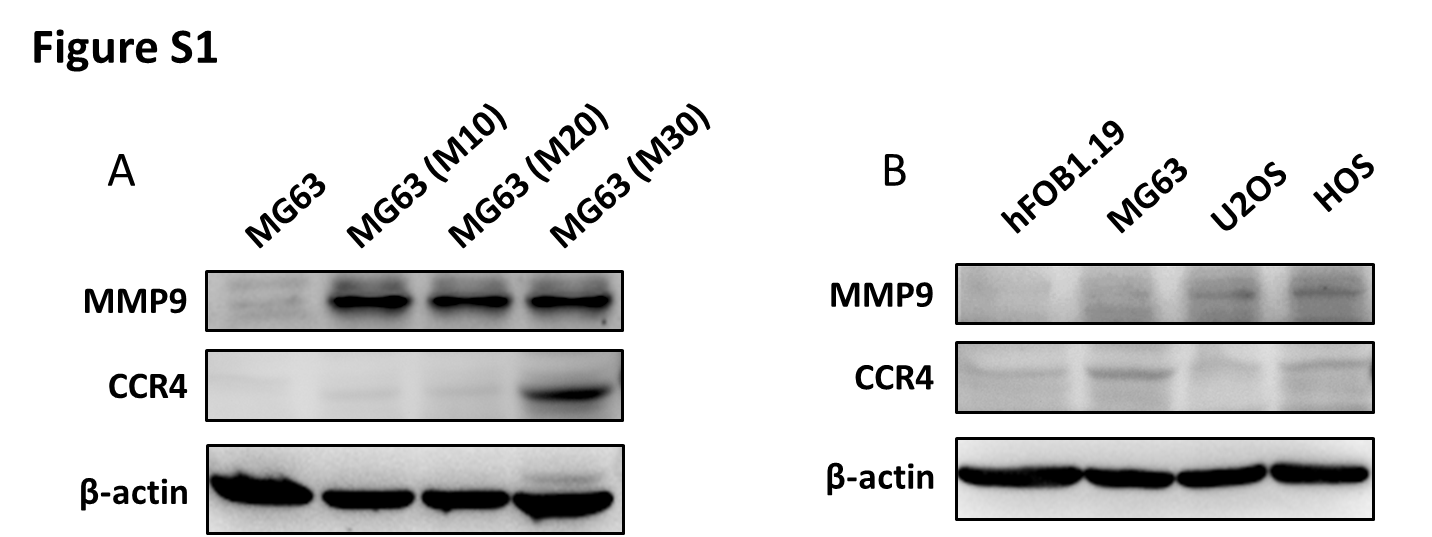


**Figure S1. Expression levels of MMP9 and CCR4 in migration-prone subclones of MG63 osteosarcoma cells, osteosarcoma cell lines and normal osteoblasts.**

Total proteins were collected from the indicated cell lines, then MMP9 and MCP-1 expression levels were evaluated by using Western blotting. β-actin was used as loading control.

**
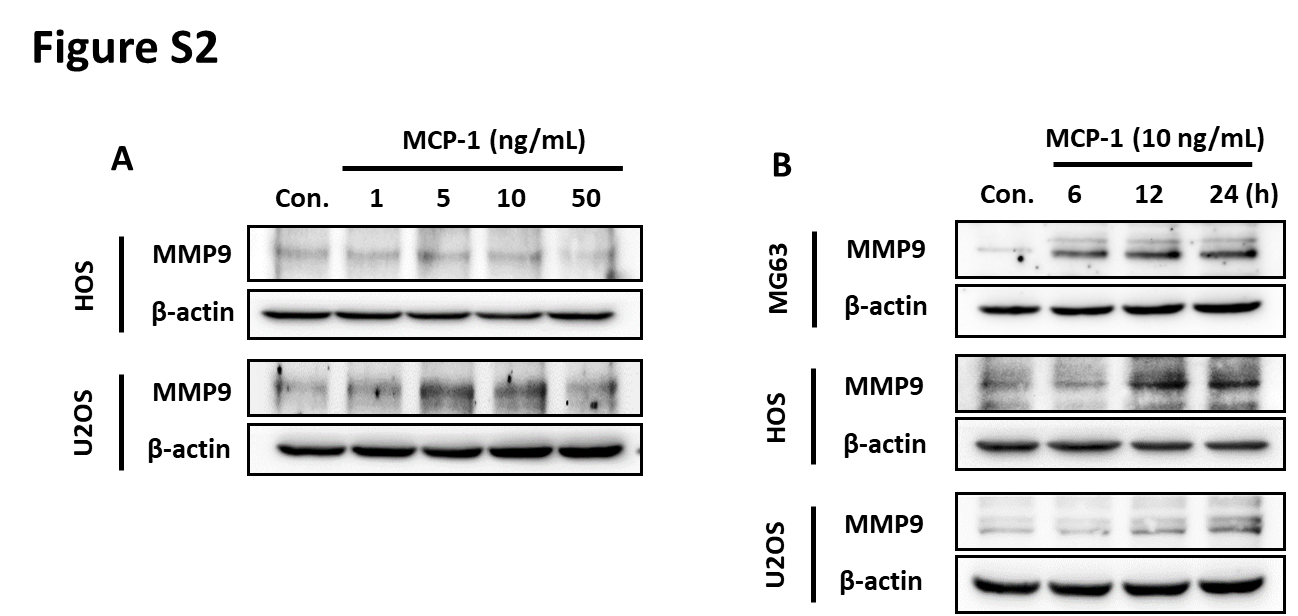
Figure S2. MPC-1 contributes to MMP-9 expression in osteosarcoma cell lines.**

(A) MMP-9 protein expression levels were monitored by using Western blotting in the HOS and U-2OS cells after stimulation with different concentrations of MCP-1 (1, 5, 10, and 50 ng/mL) for 24h. (B) The osteosarcoma cells (MG63, HOS and U2OS) were incubated with MCP-1 (10 ng/mL) for different time intervals (0, 6, 12, 24 h), followed by investigation of MMP-9 expression by using Western blotting.

**Figure S3. MCP-1 promotes osteosarcoma cells migration through CCR2,** **c-Raf, MAPK and AP-1 signal pathways.**
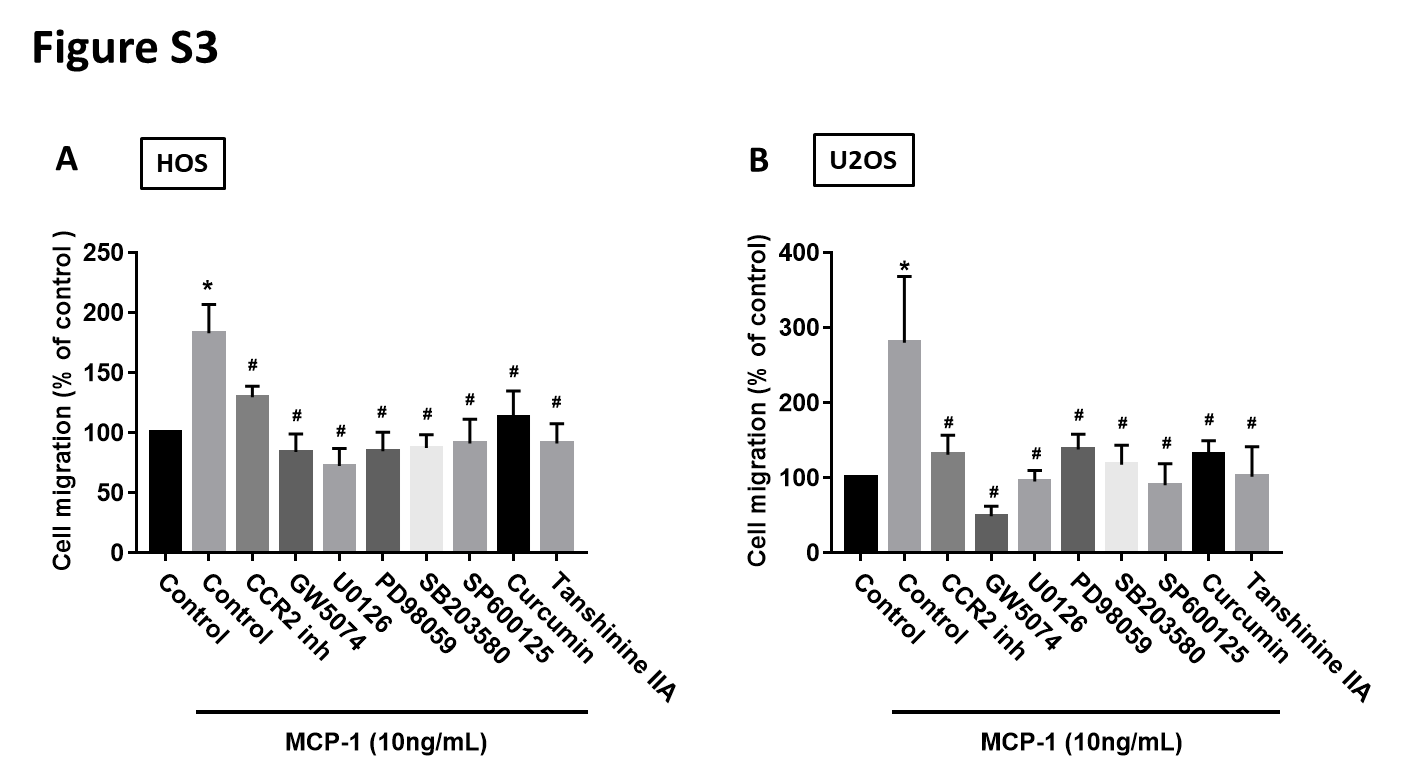


The osteosarcoma cells (HOS and U2OS) were pretreated with indicated inhibitors (CCR2 inh, 400 nM; GW5074, 5 μM; U0126, 10 μM; PD98059, 10 μM; SB203580, 10 μM; SP600125, 10 μM; curcumin, 1 μM and tanshinone IIA, 5 μM) for 30 min, followed by MCP-1 stimulation for 24 h. The cell migration assays were conducted after 24 h later. Results are expressed as mean ± SEM, n=4. *p < 0.05 compared with control group; #p < 0.05 compared with the MCP-1-treated group.
